# Comparative Analysis of BTK Inhibitors and Mechanisms Underlying Adverse Effects

**DOI:** 10.3389/fcell.2021.630942

**Published:** 2021-03-11

**Authors:** H. Yesid Estupiñán, Anna Berglöf, Rula Zain, C. I. Edvard Smith

**Affiliations:** ^1^Department of Laboratory Medicine, Clinical Research Center, Karolinska Institutet, Karolinska University Hospital, Huddinge, Sweden; ^2^Departamento de Ciencias Básicas, Universidad Industrial de Santander, Bucaramanga, Colombia; ^3^Centre for Rare Diseases, Department of Clinical Genetics, Karolinska University Hospital, Stockholm, Sweden

**Keywords:** ibrutinib, acalabrutinib, zanubrutinib, atrial fibrillation, infection, rash, diarrhoea, X-linked agammaglobulinemia

## Abstract

The cytoplasmic protein-tyrosine kinase BTK plays an essential role for differentiation and survival of B-lineage cells and, hence, represents a suitable drug target. The number of BTK inhibitors (BTKis) in the clinic has increased considerably and currently amounts to at least 22. First-in-class was ibrutinib, an irreversible binder forming a covalent bond to a cysteine in the catalytic region of the kinase, for which we have identified 228 active trials listed at ClinicalTrials.gov. Next-generation inhibitors, acalabrutinib and zanubrutinib, are approved both in the United States and in Europe, and zanubrutinib also in China, while tirabrutinib is currently only registered in Japan. In most cases, these compounds have been used for the treatment of B-lymphocyte tumors. However, an increasing number of trials instead addresses autoimmunity and inflammation in multiple sclerosis, rheumatoid arthritis, pemphigus and systemic lupus erythematosus with the use of either irreversibly binding inhibitors, e.g., evobrutinib and tolebrutinib, or reversibly binding inhibitors, like fenebrutinib. Adverse effects (AEs) have predominantly implicated inhibition of other kinases with a BTKi-binding cysteine in their catalytic domain. Analysis of the reported AEs suggests that ibrutinib-associated atrial fibrillation is caused by binding to ERBB2/HER2 and ERBB4/HER4. However, the binding pattern of BTKis to various additional kinases does not correlate with the common assumption that skin manifestations and diarrhoeas are off-target effects related to EGF receptor inhibition. Moreover, dermatological toxicities, diarrhoea, bleedings and invasive fungal infections often develop early after BTKi treatment initiation and subsequently subside. Conversely, cardiovascular AEs, like hypertension and various forms of heart disease, often persist.

## BTK, B-Lymphocyte Development and X-Linked Agammaglobulinemia

The understanding of the B-cell receptor (BCR) signaling pathway led to the deciphering of the central role of Bruton’s tyrosine kinase (BTK) and the importance of its inhibition as an effective strategy for the treatment of B-cell malignancies (Herman et al., 2011; Smith, 2017; Pal Singh et al., 2018; Lucas and Woyach, 2019). BTK is a non-receptor protein-tyrosine kinase that belongs to the TEC family of kinases. Upon BCR stimulation BTK gets phosphorylated by the SRC-family kinase LYN, and activated BTK phosphorylates its substrate, the downstream molecule phospholipase C-γ2, which results in an increased level of intracellular calcium and activation of transcription factors involved in B-cell proliferation, differentiation and survival (Smith et al., 2001). BTK is expressed in all hematopoietic cells such as macrophages, neutrophils and mast cells, with the exception of T- and plasma cells (de Weers et al., 1993; Smith et al., 1994). Mutations in the *BTK* gene in humans cause X-linked agammaglobulinemia (XLA) (Bruton, 1952; Vetrie et al., 1993), which is a primary humoral immunodeficiency characterized by an arrest in the B-cell development, at the transition between the pro-B to the pre-B cell stage, with almost total lack of immunoglobulin production (Campana et al., 1990; Del Pino Molina et al., 2019). The central role of BTK is not restricted to normal B-cells; this kinase is also important for the proliferation, migration and survival of malignant B-cells (De Rooij et al., 2012). Therefore, BTK binding and obstruction of proliferative and pro-survival signals caused by impaired adhesion properties is assumed to be the main mechanism of BTK inhibitors (Nore et al., 2000; Bernal et al., 2001; De Rooij et al., 2012).

## Ibrutinib First-In-Class BTK Inhibitor

Ibrutinib (Imbruvica^®^), the first-in-class BTK inhibitor (BTKi), is an irreversible binder, which has revolutionized the therapeutic landscape for B-cell malignancies (Honigberg et al., 2010; Advani et al., 2013). It is the most studied, and first, BTKi approved by the United States Food and Drug Administration (FDA) and the European Medicines Agency (EMA). Approval includes the following indications: pretreated adults with mantle cell lymphoma (MCL), previously treated, or untreated, chronic lymphocytic leukemia/small lymphocytic leukemia (CLL/SLL) and Waldenström macroglobulinemia (WM). Ibrutinib is also approved by the FDA for previously treated marginal zone lymphoma (MZL) patients and chronic graft-versus-host disease (cGVHD) (Table 1; FDA, 2020).

**TABLE 1A:**
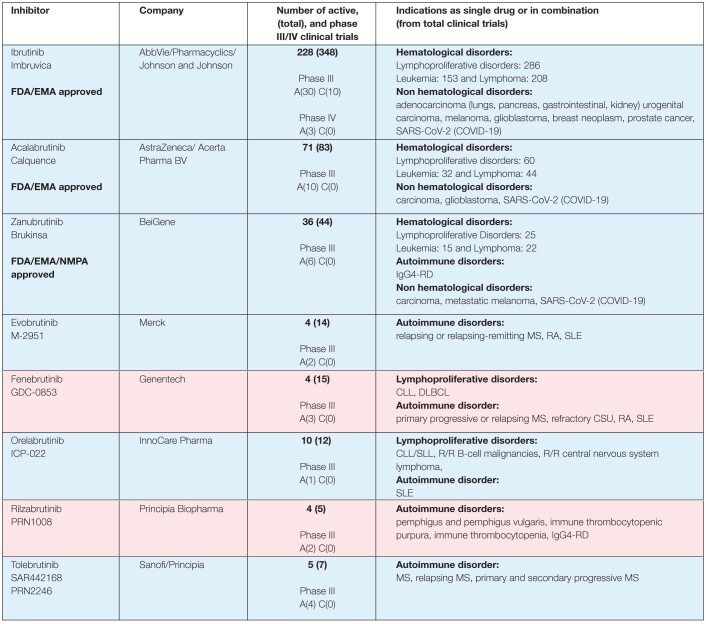
BTK inhibitors in phase III clinical trials.

*Covalent BTKis, blue background; non-covalent BTKis, light orange background and BTKis with unknown mechanism of binding white; A, active; C, completed; CLL, chronic lymphocytic leukemia; CSU, chronic spontaneous urticaria; DLBCL, diffuse large B-cell lymphoma; EMA, European Medicines Agency; FDA, Food and Drug Administration; IgG4-RD, IgG4-related disease; MCL, mantle cell lymphoma; MS, multiple sclerosis; NHL, non-Hodgkin’s lymphoma; NMPA, National Medical Products Administration – China; PMDA, Pharmaceuticals and Medical Devices Agency – Japan; R/R, relapsed/refractory; RA, rheumatoid arthritis; SLE, systemic lupus erythematosus; SLL, small lymphocytic lymphoma; TN, treatment naïve; WM, Waldenström’s macroglobulinemia.*

**TABLE 1B:**
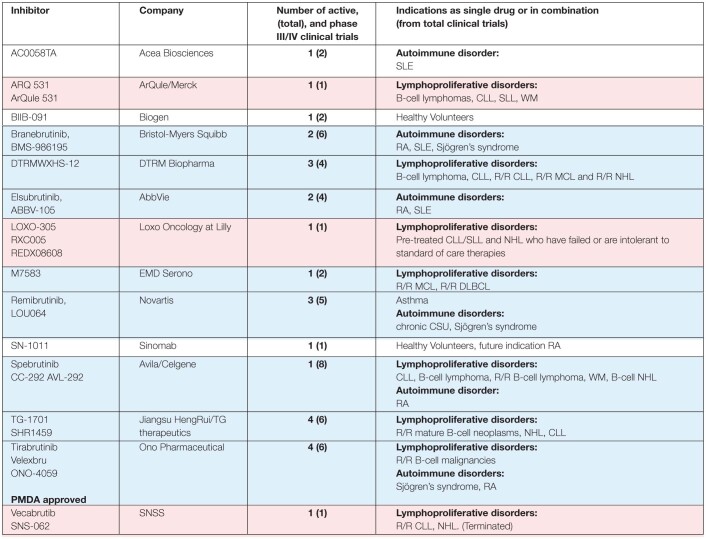
BTK inhibitors, other than those presented in Table 1A, in phase I/II clinical trials.

Ibrutinib is generally well tolerated with rapid and durable response. However, off-target binding, usually associated with treatment-emergent adverse effects (AEs) such as rash, diarrhoea, bleedings, infections and atrial fibrillation (AF) leads to treatment discontinuation in a substantial number (9 – 23%) of patients in clinical studies (Byrd et al., 2015; Jain et al., 2015, 2017; Mato et al., 2018; Munir et al., 2019). In three different studies from community practice, 23% out of 447, 41% out of 616 and 49% out of 95 patients discontinued ibrutinib treatment (Sharman et al., 2017; Mato et al., 2018; Winqvist et al., 2019).

## Next-Generation BTKi

Next-generation inhibitors with different binding-profiles and improved selectivity were developed to decrease the observed ‘off-target’ effects of ibrutinib (Liu et al., 2018). Acalabrutinib (Calquence^®^) and zanubrutinib (Brukinsa^®^) are the most studied next-generation BTKis, and show potential in both preclinical and clinical studies with improved selectivity and with less AEs than ibrutinib (Byrd et al., 2016; Wu et al., 2016; Tam et al., 2020a). Acalabrutinib is FDA- and EMA approved for adults with previous treated, or untreated, CLL/SLL and only FDA approved for pretreated MCL patients. Zanubrutinib got accelerated approval for MCL, EMA approval for pretreated WM patients, and National Medical Products Administration (NMPA) approval in China for CLL/SLL and pretreated MCL patients (Table 1; FDA, 2019a, b). Tirabrutinib (Velexbru^®^) is approved in Japan by the Pharmaceuticals and Medical Devices Agency (PMDA) for treatment of recurrent or refractory primary central nervous system lymphoma and also received supplemental approval for WM and lymphoplasmacytic lymphoma (Dhillon, 2020).

## Resistance Mutations

Unfortunately, ∼60% of ibrutinib long-term treated patients eventually acquire resistance to covalent inhibitors, caused by the development of clones that most frequently carry a mutated cysteine (C481) in the ibrutinib binding site. The commonest cause of resistance is the C481 to serine substitution in BTK (Woyach et al., 2014, 2017; Hamasy et al., 2017; Quinquenel et al., 2019). To overcome this limitation, non-covalent binding compounds such as Fenebrutinib (GDC-0853), ARQ 531 (ArQule 531) or LOXO-305 (RXC005, REDDX08608) represent an alternative and were found to be effective when C481 was substituted by serine or arginine, whereas other covalent inhibitors also lose potency against C481 mutants (Johnson et al., 2016; Reiff et al., 2018a, b; Bond and Woyach, 2019; Naeem et al., 2019).

## Clinical Trials for the Increasing Number of BTKis

This is a highly expanding and competitive field. At the time of writing this review, 22 covalent and noncovalent BTKis are already in clinical trials (Table 1). For ibrutinib, 228 active clinical trials are registered at the ClinicalTrials.gov and the majority address hematological disorders, such as leukemia or lymphoma. The clinical studies of non-hematological disorders, registered for ibrutinib are for adenocarcinoma, carcinoma, melanoma, glioblastoma, breast neoplasm, prostate cancer and SARS-CoV-2 (COVID-19). For acalabrutinib and zanubrutinib, 71 and 36 active clinical trials are found, respectively, of which 60 and 25 address lymphoproliferative disorders (Table 1).

Furthermore, several phase III clinical trials are ongoing, such as NCT02477696 in CLL comparing acalabrutinib versus ibrutinib, and NCT03053440 (ASPEN) / NCT03734016 (ALPINE) analyzing the effect of zanubrutinib versus ibrutinib in WM and CLL, respectively. Clinical trials combining BTKis with other drugs such as anti-CD20 or BCL2 inhibitors are also ongoing e.g., NCT03701282, NCT03580928, NCT03824483 and combining a BTKi with an anti-apoptotic drug may have curative potential (Jones et al., 2018).

Five other BTKis are also in active phase III clinical trials: evobrutinib (M-2951), fenebrutinib (GDC-0853), tolebrutinib (SAR442168, PRN2246), orelabrutinib (ICP-022) and rilzabrutinib (PRN1008) indicated for patients with autoimmune and lymphoproliferative disorders. Rilzabrutinib is of particular interest for certain indications, since it efficiently crosses the blood-brain barrier (Table 1; Ramadass et al., 2020). Some other BTKis, like poseltinib (HM71224), CT-1530, BIIB-068, TAK-020 or GDC-0834, were stopped, discontinued or no further development was reported.

Other compounds are considered to be dual inhibitors, such as EGFR/BTK (olafertinib, DZD-9008 and AC0010/abivertinib) or FLT3/BTK (CG-806) inhibitors (Xu et al., 2016; Huang et al., 2019; Kim et al., 2019; Ramadass et al., 2020). Yet others are used in concert such as in the case of ABBV-599, which is the combination of the BTKi ABBV-105, with JAK1 inhibitor ABT-494 (phase II clinical trial NCT03682705).

## Pre-Clinical Development of New BTKis

Several new BTKis are also in pre-clinical development, among them, the chimeric targeting molecule NRX049, which is first in its class. It belongs to the PROteolysis TArgeting Chimera (PROTAC) type of degrading compounds. PROTACs consist of two covalently linked protein-binding moieties: one that binds to a target protein meant for degradation, and another, which recruits an E3 ubiquitin ligase. NRX049 seems to be highly effective in CLL cells and experimental *in vivo* studies are ongoing in patient-derived xenograft models (Zhang et al., 2019).

## Assaying the Selectivity of BTKis

In Table 2 we summarize the available data on the activity of inhibitors in active clinical trials. For six of the inhibitors included in Table 1 information about binding is not in the public domain. For some of the inhibitors included in Table 2 only limited data is available, either from biochemical kinase assays or percentage of inhibition, as for the covalent BTKis evobrutinib, TG-1701 (SHR1459), M7583 and branebrutinib (BMS-986195) and the non-covalent BTKis ARQ531 (ARQule531) and LOXO-305.

**TABLE 2:**
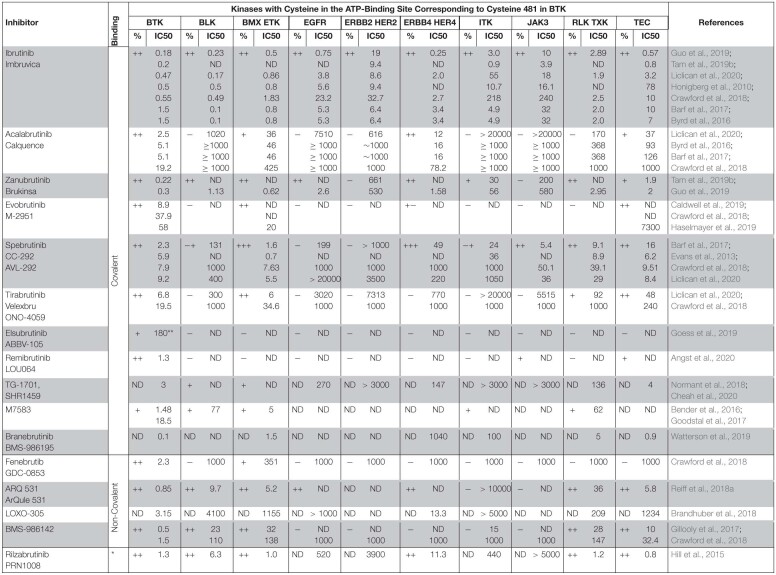
Percentage of inhibition and IC50 values reported for BTK inhibitors.

(%) Predominant percentage of inhibition: (–) < 50% inhibition; (+) = or > 50% inhibition; (++) = or > 80% inhibition; IC50 (nM) values, obtained from in vitro biochemical kinase assay, are shown from lower (top) to higher number (bottom); ND: no data available; * non-covalent reversible; ** value obtained by activity assay using recombinant protein including only the kinase domain.

Ibrutinib binds covalently to the C481 thiol group in the ATP-binding site of the kinase domain of BTK, and also in varying degree to the corresponding site in nine other kinases (Honigberg et al., 2010; Byrd et al., 2013). Four of them are TEC family kinase members: ITK, interleukin-2-inducible T-cell kinase; TEC, tyrosine kinase expressed in hepatocellular carcinoma; BMX, bone marrow-expressed kinase; and RLK/TXK, resting lymphocyte kinase/ T and X cell expressed kinase (Smith et al., 2001). Three of these belong to the EGFR family of kinases: EGFR, Epidermal Growth Factor Receptor; ERBB2/HER2 Human Epidermal Growth Factor Receptor 2; and ERBB4/HER4 Human Epidermal Growth Factor Receptor 4, while the remaining two tyrosine kinases are B-lymphoid kinase (BLK), a member of the SRC family of kinases, and Janus kinase 3 (JAK3) (Honigberg et al., 2010; Byrd et al., 2013; Berglöf et al., 2015).

Various kinase activity assays are used to determine the selectivity of the inhibitors. The diversity of the approaches and the different results obtained from biochemical and cellular assays illustrate the complexity when comparing inhibition profiles of BTKis. For example, IC50 values obtained from *in vitro* activity measurements do not always correlate with the kinase selectivity of the compound when examined in a cellular context. Therefore, comparative studies are of particular interest, where several inhibitors are tested in parallel using the same assay (Table 2; Byrd et al., 2016; Barf et al., 2017; Crawford et al., 2018; Liclican et al., 2020). There are a number of *in vitro* biochemical kinase assays used in the cited reports such as: LanthaScreen (TR-FRET), Z-LYTE and IMAP (FP or TR-FRET). However, these tests have limitations, such as the time-dependent effect during the biochemical determination of the IC50 value for covalent inhibitors (Angst et al., 2020). Furthermore, compound screening using kinase panels and determination of binding constants are used to evaluate the BTKi selectivity (Table 2).

For several of the reviewed BTKis, the reported IC50 data for kinases other than BTK are highly variable, e.g. the acalabrutinib biochemical IC50 values for TEC vary from 37 to 1000 nM (Byrd et al., 2016; Barf et al., 2017; Crawford et al., 2018; Angst et al., 2020; Liclican et al., 2020). Further examples are: spebrutinib inhibition of ITK has been reported as < 40 nM or ≥ 1000 nM (Evans et al., 2013; Byrd et al., 2016; Barf et al., 2017; Crawford et al., 2018; Liclican et al., 2020) and for tirabrutinib, IC50 data presented for RLK/TXK differ by more than 10-fold (Byrd et al., 2016; Crawford et al., 2018; Liclican et al., 2020).

Moreover, the data from biochemical assays frequently do not correlate with cellular test results. For example, IC50 values for acalabrutinib from biochemical assays reported TEC inhibition at 37, 93 as well as 126 nM (Table 2). A TEC phosphorylation assay using human platelets shows that acalabrutinib does not inhibit TEC and that a completely non-pharmacological concentration of > 1000 nM inhibits even less than 25% of TEC phosphorylation (Byrd et al., 2016). Zanubrutinib suppresses TEC based on the IC50 values from biochemical assays (around 2 nM), while the value for ibrutinib is higher (3.2–78 nM) (Table 2) and it is well-established that ibrutinib inhibits this kinase. The data from a cellular assay for TEC phosphorylation suggest that zanubrutinib is more selective than ibrutinib with preferential inhibition of BTK over TEC, although different assays were used in this comparison (Guo et al., 2019).

A similar discrepancy is also observed for zanubrutinib when ITK inhibition is evaluated in Jurkat leukemia cells. Blocking of phosphorylation in the ITK substrate PLCG1 requires 3477 nM of zanubrutinib (Guo et al., 2019; Tam et al., 2019b), whereas a much lower IC50 of 30 and 56 nM is reported in the biochemical assays (Table 2).

In contrast, when acalabrutinib or spebrutinib are *in vitro* evaluated against EGFR (Barf et al., 2017) or EGFR/JAK3 kinases, respectively (Evans et al., 2013), results from biochemical and cellular data correlate, demonstrating that neither kinase inhibits EGFR and that only spebrutinib inhibits JAK3 kinase activity. Apart from biochemical and cellular assays, we have recently generated a knock-in mouse, which carries a cysteine 481 to serine mutation in BTK, enabling adequate *in vivo* off-target analysis for all irreversible inhibitors (Estupiñán et al., 2020).

## Brief Introductory Overview to Adverse Effects

In this survey we aim to correlate inhibition profiles of BTKis with reported AEs. However, there are several limitations. At present clinical data from long-term clinical trials or real-world data are only available for a few of the inhibitors, namely for ibrutinib (Mato et al., 2018; Munir et al., 2019), acalabrutinib (Yazdy et al., 2019; Sharman et al., 2020) and zanubrutinib (Tam et al., 2019b, 2020a), but the follow-up time varies among the studies. Thus, the available dataset on AEs is limited for several of the inhibitors such as tirabrutinib (Sekiguchi et al., 2020) or fenebrutinib (Cohen et al., 2020), due to low patient enrollment, short-term trials or that the clinical data have not been published as yet.

Apart from binding to the group of tyrosine kinases with a cysteine in the catalytic pocket, ibrutinib also tethers reversibly to many kinases that lack cysteine in their binding site. Thus, it interacts reversibly with e.g., the SRC-family regulatory kinase, C-terminal SRC kinase (CSK), and the SRC family kinases FGR and HCK in low nanomolar range, comparable with the irreversible binding to BTK (IC50 0.5 nM) (Honigberg et al., 2010).

Ibrutinib potently inhibits BTK causing reduced BCR signaling (Honigberg et al., 2010), but it also targets many other cellular processes through the roles of BTK outside of the BCR (Nore et al., 2000). The direct inhibition of other kinases impacts upon normal processes in T lymphocytes (no contribution of BTK) as well as macrophages and platelets (both cell types express BTK and also other kinases interacting with BTKis) (Feng et al., 2015; Byrd et al., 2016; Barf et al., 2017; Nicolson et al., 2018; Estupiñán et al., 2020).

Off-BTK effects in B- or non-B-cells have a therapeutic potential and they may also account for the observed AEs, since those cannot be explained by BTK inhibition alone. As will be discussed in greater detail below, binding to BTK in B-cell malignancies has a treatment effect, while inhibition of this kinase by BTKi in macrophages or neutrophils was suggested to impair the anti-fungal response (Fiorcari et al., 2020). However, it should be pointed out that in X-linked agammaglobulinemia, defined by non-functional BTK, a similar overt propensity for *invasive* fungal infections is not seen, nor are there increased bleedings, skin manifestations, diarrhoeas or cardiovascular disease (Smith and Berglöf, 1993; Ochs and Smith, 1996). Several of the inhibitors bind to TEC, which is expressed in CLL cells at similar levels as BTK, suggesting that concomitant binding to both of these kinases could contribute to the anti-tumor effect (de Bruijn et al., 2017). On the other hand, binding to both BTK and TEC in platelets is related to bleedings, an adverse effect of BTKi treatment (Levade et al., 2014; Kamel et al., 2015; Nicolson et al., 2018).

Another point to consider for AEs relates to the disease which is treated, since they could differ between lymphoid malignancy and autoimmunity. It is well-known that B-lymphoid neoplasms can suppress the formation of essentially all components of the hematopoietic system, including platelets and granulocytes. This also means that when BTKi-induced reduction of the tumor burden occurs, this could have a beneficial effect on the production of both thrombocytes and granulocytes. This may also contribute to that certain AEs are transient, since initially bleedings and neutropenia may originate from the combined effect of BTKis interfering with signaling pathways together with the already ongoing suppressed hematopoiesis caused by the tumor burden. Following treatment, the negative impact of the tumor burden on hematopoiesis will subside. Based on this we predict that, while the effects of BTKis in autoimmunity are considerably less studied, some of the AEs observed in hematopoietic malignancies will only occur in this patient population.

## Ibrutinib Treatment Increases the Risk for Development of Cardiovascular Disease, Including Atrial Fibrillation and Hypertension

Although cancer-related inflammation remains a risk factor for AF (Chu et al., 2019), AF is not increased in the CLL patient population. AF has, however, been reported as a relatively common adverse effect of ibrutinib (Byrd et al., 2013; McMullen et al., 2014; Burger et al., 2015; Baptiste et al., 2019). Hence, treated patients have a higher risk of developing AF than age-matched, healthy individuals or CLL patients who do not receive any BTKi (Ganatra et al., 2018; Baptiste et al., 2019; Salem et al., 2019). Ventricular arrhythmias, conduction disorders and hypertension, have also been observed (Salem et al., 2019) and in mice, the inducibility of atrial and ventricular arrhythmia increases after ibrutinib intake (Tuomi et al., 2018). While this needs further investigation, there is evidence that the risk to develop cardiovascular AEs increases over time (Archibald et al., 2020) and during a 7-year follow-up of ibrutinib therapy, hypertension was sustained (Byrd et al., 2020a).

In more than 500 patients treated with ibrutinib for malignancies from 2009 through 2016 hypertension rates were studied (Dickerson et al., 2019). More than 3/4 patients developed new or worsened high blood pressure over a median of 30 months. Hypertension was associated with increased major adverse cardiovascular events including arrhythmia, myocardial infarction, stroke, heart failure, and cardiovascular death. Antihypertensive therapy reduced the cardiovascular complications.

Interestingly, there are differences between treatment-naïve and the relapsed or refractory (R/R) population. Results from a real-world analysis in the United States of 616 ibrutinib-treated patients, with a median follow-up of 17 months, show atrial fibrillation in 25% of patients front-line treated with ibrutinib, and in 12 % of the relapsed/refractory population (Mato et al., 2018). Furthermore, the results from company-sponsored clinical trials and real-world data differ as mentioned above. Based on the results from clinical trials summarized in Table 3, significantly fewer cases of AF were recorded for patients receiving zanubrutinib- (0–5% *all grades*; < 2% for ≥ *grade 3*) or acalabrutinib- (1–7% *all grades*; < 3% for ≥ *grade 3*) than ibrutinib (10–17% *all grades*; 1.6 – 9% for ≥ *grade 3*). Thus, treatment with zanubrutinib and acalabrutinib, results in a diminished number of AF cases. Although the results from a comparative trial between acalabrutinib and ibrutinib are not yet available, a phase III randomized study with 533 R/R CLL patients is ongoing for acalabrutinib versus ibrutinib monotherapy (NCT02477696).

**TABLE 3:**
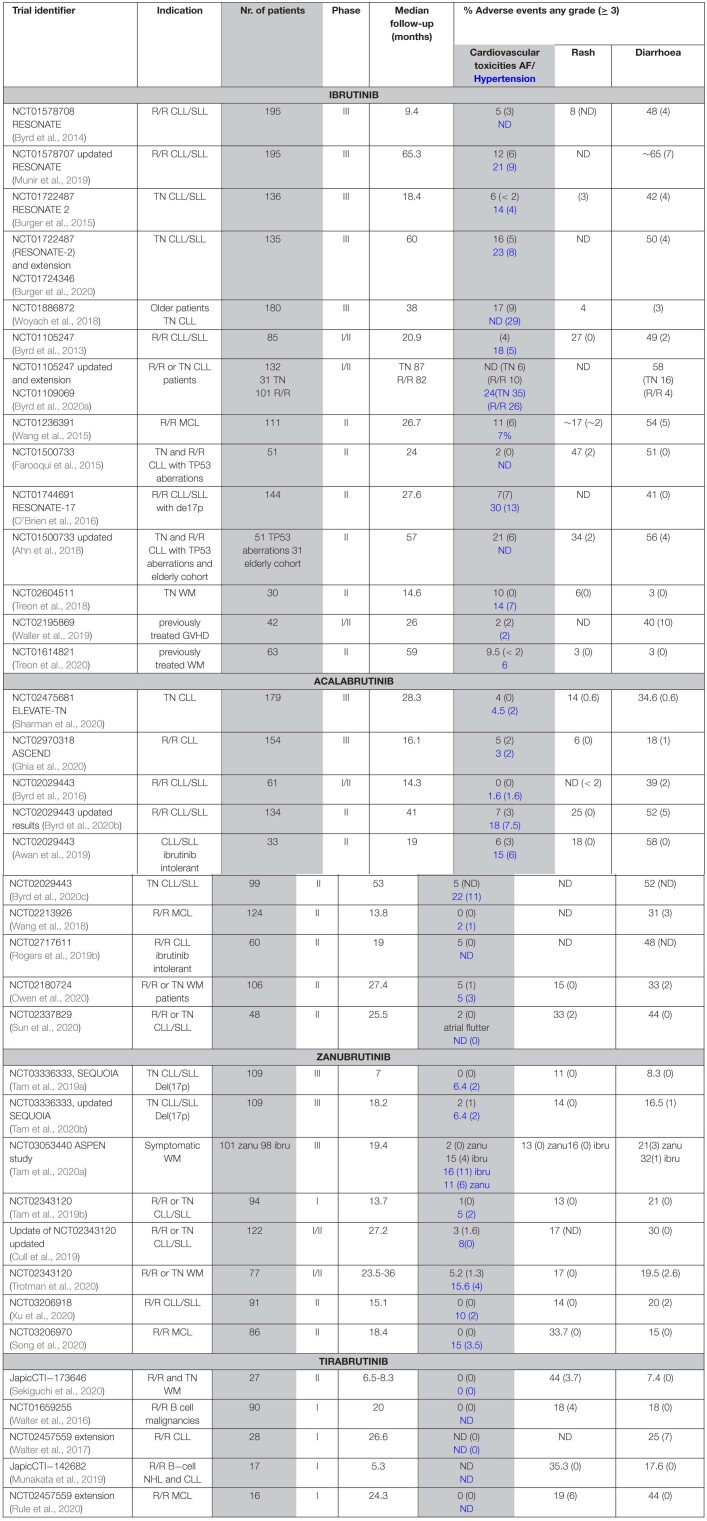
Atrial fibrillation, rash and diarrhoea in patients treated with BTK inhibitors.

*AF, Atrial fibrillation; CLL, chronic lymphocytic leukemia; GvHD, Graft versus host disease; MCL, mantle cell lymphoma; ND, no data available; NHL, non-Hodgkin lymphoma; H, hypertension; R/R, relapsed/refractory; SLL, small lymphocytic lymphoma; TN, treatment naïve; WM, Waldenström’s macroglobulinemia.*

Moreover, the results of a head-to-head phase III multicenter study (ASPEN study) comparing zanubrutinib versus ibrutinib in patients with WM are already at hand. Both AF and hypertension were reported at greater frequency for ibrutinib, compared with zanubrutinib at a median follow-up of 19.4 months. Thus, AF was reported at a ≥ 10% higher incidence among ibrutinib treated patients (*all grades* 15/98 versus 2/101). There were no ≥ *grade 3* cases of AF in the zanubrutinib group, while in the ibrutinib group 4/98 were observed (Tam et al., 2020a). Another phase III, randomized, open-label (ALPINE) study comparing zanubrutinib with ibrutinib in approximately 600 patients with R/R CLL/SLL is ongoing and will provide further comparison. The first patient was dosed in November 2018. Based on enrollment prediction, the study duration is estimated to be 51 months (Hillmen et al., 2020).

## Cardiovascular Targets of BTKis

It has been suggested that AF may be partially mediated by decreased phosphatidylinositol-3-kinase (PI3K)-AKT signaling in cardiomyocytes due to BTK and TEC inhibition (McMullen et al., 2014). While we are unaware of any conclusive evidence demonstrating BTK expression in the heart, TEC has been detected in rat neonatal cardiomyocytes (Bony et al., 2001) and in adult mouse cardiac myocytes, where it is implicated in myocardial ischaemia (Zhang et al., 2010). It was also shown that blocking ERBB2/HER2, which is another kinase with the potential for irreversible ibrutinib-binding (Table 2), results in cardiomyocyte dysfunction and reduced heart contractile efficiency. Such an AE was observed in patients with breast cancer treated by specific HER2 inhibitors, unrelated to BTKi (Albini et al., 2011). It was also shown that a conditional ERBB2/HER2 mutation in ventricular cardiomyocytes leads to impaired cardiac conduction (Özcelik et al., 2002) and this kinase is required for atrial electrical activity during development (Tenin et al., 2014).

Moreover, another member of the EGFR family, ERBB4/HER4, is also expressed in the heart. To this end, HER4 – HER2 heterodimerization with subsequent downstream signaling, including the PI3K−AKT pathway, is important for normal heart physiology (Milano et al., 2014). Whereas BMX, which is an irreversible BTKi-binding kinase, was shown to play a role in the cardiovascular system (Holopainen et al., 2012), we do not favour its involvement in AF owing to its BTKi interaction pattern, which is not related to the frequency of AF (Table 3).

Thus, there are several possible explanations for the fact that ibrutinib’s interaction with target proteins, other than BTK, could cause cardiac dysfunction, including AF. We favour the idea of an off−BTK target mechanism, as proposed (Berglöf et al., 2015). Based on the results presented in Table 2, we notice that ibrutinib inhibits all the remaining kinases implicated in causing cardiovascular side effects, namely HER2, HER4 and TEC, whereas acalabrutinib inhibits HER4, and only slightly TEC, but not HER2. Conversely, zanubrutinib seems to inhibit TEC and HER4, but not HER2, while tirabrutinib inhibits TEC but neither HER2 nor HER4.

## Potential BTKi Off-Targets Causing AF

For ibrutinib, the binding to HER2 has been previously implicated as a candidate for cardiovascular AEs (Salem et al., 2019). Furthermore, ibrutinib, zanubrutinib and tirabrutinib all inhibit TEC, whereas acalabrutinib does not. This suggests that binding to, and inhibiting TEC is not of importance, since there seems to be no profound differences between zanubrutinib and acalabrutinib for causing cardiovascular toxicity (Table 3). The simultaneous binding to HER2 and HER4 is unique to ibrutinib and could account for the decisive difference, since zanubrutinib and acalabrutinib differ in this regard, both being only rarely associated with AF. Based on this information it is not possible to discriminate between whether targeting HER2 is critical, or alternatively, if simultaneous targeting of HER2 and HER4 is needed. We also cannot exclude the possibility that other targets than kinases are causing AF. However, current evidence suggests that tyrosine kinases binding irreversibly to BTKis, and being expressed in the heart, are strong candidates and this mechanism may be sufficient to explain this AE.

## Increased Risk for Bleedings Under BTKi Treatment

Bleeding is another well-known, ibrutinib-associated AE. In an integrated analysis of 15 ibrutinib clinical trials for lymphoid malignancies, including 4 randomized controlled trials, it was shown that bleeding of any-grade has an overall incidence of 40% (Brown et al., 2019). Around 4% of treated patients develop major haemorrhage, but in only 1% of all treated cases it leads to ibrutinib discontinuation (Brown et al., 2019).

Increased risk of haemorrhage is associated with ibrutinib treatment in the presence or absence of thrombocytopenia (Byrd et al., 2013; de Weerdt et al., 2017; Shatzel et al., 2017; Brown, 2018; Gribben et al., 2018; Stephens and Byrd, 2019) and when ibrutinib is combined with antiplatelet or anticoagulation medications (Mock et al., 2018). These are usually used as therapy to reduce the risk of thromboembolism in patients with AF (Brown et al., 2019), which, as mentioned, is overrepresented among ibrutinib-treated patients.

The highest risk of haemorrhage occurs in the first months of treatment, suggesting that both disease and treatment-related factors influence the severity of this AE (Ysebaert et al., 2014; Lipsky et al., 2015; Brown et al., 2019; Dmitrieva et al., 2020). Furthermore, patients with B-cell malignancies have an intrinsic risk of bleeding (Gifkins et al., 2015).

Low-grade bleeding events are usually not associated with thrombocytopenia, suggesting an impaired platelet function as the cause of the AE as reviewed (Berglöf et al., 2015). However, Dmitrieva et al. (2020) investigated the platelet function in 50 CLL and 16 MCL patients and their results suggest that ibrutinib-dependent bleeding in CLL patients requires a setting of three mechanisms. Most important was a decreased platelet count prior ibrutinib treatment.

Bruton’s tyrosine kinase and TEC are both involved in collagen-induced platelet activation and ibrutinib binding to both kinases has been associated with impaired coagulation (Quek et al., 1998; Oda et al., 2000; Levade et al., 2014). Furthermore, platelets from treated patients that experienced bleedings show low aggregation response to collagen and unaffected aggregation response to ADP, thrombin or thromboxane A2 (Levade et al., 2014; Kamel et al., 2015). Reduced platelet adhesion to von Willebrand Factor-coated surfaces has been also observed (Levade et al., 2014). Interestingly, both the defect in the collagen response and the reduction in the adhesion were reversed after treatment interruption (Levade et al., 2014; Kamel et al., 2015).

Bleedings caused by ibrutinib cannot only be associated with inhibition of both BTK and TEC, since this AE has also been found after treatment with more selective BTKis, such as acalabrutinib and zanubrutinib (Byrd et al., 2020b; Ghia et al., 2020; Owen et al., 2020; Sharman et al., 2020; Song et al., 2020; Sun et al., 2020; Tam et al., 2020a; Trotman et al., 2020; Xu et al., 2020). *In vitro* experiments in human platelets showed that acalabrutinib does not inhibit TEC (Byrd et al., 2016; Nicolson et al., 2018), which would suggest a reduced number of cases with bleeding. However, bleeding is still observed using acalabrutinib at variable frequencies, *any-grade* 26–58% and major bleeding 1–5% (Byrd et al., 2020b; Ghia et al., 2020; Owen et al., 2020; Sharman et al., 2020; Sun et al., 2020). This is also the case for zanubrutinib (Guo et al., 2019), where 4.4–66% of *any-grade* and 0.3–2.2% of major bleedings were observed in treated patients (Song et al., 2020; Tam et al., 2020a; Trotman et al., 2020; Xu et al., 2020). In addition, as mentioned, data from a cellular assay for TEC phosphorylation suggest that zanubrutinib is less prone to interfere with TEC as compared to ibrutinib (Guo et al., 2019).

In a comparative study, where the effect of ibrutinib and zanubrutinib on platelet function was evaluated, it was found that ibrutinib is not only involved in the inhibition of collagen response and platelet activation, but also, in contrast to zanubrutinib, in reduced expression of GPIbα, GPIX, and integrin αIIbβ3. *Ex vivo* thrombus formation on type I collagen during arterial flow was reduced in CLL patients treated with ibrutinib when compared with zanubrutinib (Dobie et al., 2019). To this end, ibrutinib has even been considered as a potential new treatment fort atherothrombosis (Busygina et al., 2019). Thus, in conclusion, while BTKis can cause bleedings, this only rarely results in treatment discontinuation and the underlying mechanism is not completely understood. The combination of a tumor burden suppressing platelet formation and impaired intracellular signaling, caused by BTKis, could be responsible for this frequently transient AE.

## Mechanisms Underlying Rash

Dermatological toxicities are among the most common AEs of ibrutinib with mostly mild to moderate intensity. Their incidence is highest during the first year of treatment (Sibaud et al., 2020). This also means that in the clinical trials with long follow up, dermatological side-effects are scarce. However, if severe, they are among AEs that lead to ibrutinib discontinuation (Sharman et al., 2017; Mato et al., 2018; Yazdy et al., 2019). In the retrospective analysis of real-world acalabrutinib-treated CLL patients, intolerant to ibrutinib, it was shown that rash led to discontinuation of ibrutinib in 10 (22%) of 46 patients. During treatment with acalabrutinib, with the median follow- up of 5 months, rash occurred in 5 (7%) of patients (Yazdy et al., 2019).

In the clinical trial NCT02029443, 24% of 33 ibrutinib-intolerant patients subsequently treated with acalabrutinib, reported rash as one of AEs leading to ibrutinib intolerance. Six percent of ibrutinib-intolerant patients had rash *≥ grade* 3. Of the recorded 8 rash events, 5 did not recur, one returned, but with a lower grade, whereas only 2 recurred with the same grade upon acalabrutinib treatment (Awan et al., 2019).

While the overall incidence of the toxicities is reduced, both acalabrutinib and zanubrutinib are associated with a range of dermatologic AEs not different from those described for ibrutinib. These include bruising and ecchymoses, panniculitis, human herpesvirus infections, cellulitis, and skin rash. As summarized in a recent review on dermatological side effects, rash occurs in 13–27% (0–3% *grade* 3) of patients treated with ibrutinib, as compared to 15–18% and 13–18% in patients receiving acalabrutinib or zanubrutinib, respectively (Sibaud et al., 2020). As presented in Table 3, 11–33% (0% *≥ grade 3*) of zanubrutinib, 6–33% (0–< 2% *≥ grade 3*) of acalabrutinib and 11–44% (0–6% *≥ grade 3*) of tirabrutinib treated patients experience rash. Rash is usually considered to be an EGFR-related toxicity in patients receiving BTKis (Lucchini et al., 2014; Kozuki, 2016). This assumption is based on the fact that dermatologic side effects are relatively common among patients treated with EGFR inhibitors (Kiyohara et al., 2013; Lucchini et al., 2014; Kozuki, 2016; Zhang et al., 2016; Hsu et al., 2018).

To this end, it was reported that cutaneous irruptions from ibrutinib resemble EGFR inhibitor-induced dermatologic AEs (Singer et al., 2019). Of potential interest is also the fact that ibrutinib increases EGFR expression in dermal fibroblasts in the HDF3CGF system (Haselmayer et al., 2019; Angst et al., 2020). EGFR kinase inhibitors erlotinib and gefitinib also cause this AE. Moreover, augmented EGFR levels in the BioMAP HDF3CGF systems seem to be common to all EGFR kinase inhibitors and correlate with skin rash (Liu et al., 2013).

Importantly, when comparing data from patients treated with ibrutinib, acalabrutinib, zanubrutinib and tirabrutinib, the correlation of EGFR inhibition with rash is not obvious. Acalabrutinib virtually does not inhibit EGFR and was shown to have at least 10 times lower affinity for EGFR when compared to ibrutinib, Table 2 (Byrd et al., 2016; Liclican et al., 2020). Tirabrutinib shows a 440-fold selectivity for BTK over EGFR (Liclican et al., 2020). In cellular assays zanubrutinib exhibits lower off-target activity against EGFR and 6-fold lower preference for EGFR when compared to ibrutinib (Guo et al., 2019; Tam et al., 2019b).

Interestingly, it has been proposed that some of these forms of rash, especially those occurring within the first 4 weeks of therapy, may be consistent with the transient hyperlymphocytosis associated with ibrutinib and caused by the initial egress of CLL cells from lymph nodes and spleen (Iberri et al., 2018). If this is the case, it could explain the fact that tumor patients treated with acalabrutinib, zanubrutinib and tirabrutinib also suffer from rash while, conversely, the involvement of EGFR inhibition is not crucial. In conclusion, based on all of these observations of skin toxicities, and especially the fact that certain BTKis essentially do not bind to the EGFR at pharmacological concentrations, other off-targets should be considered.

## Mechanisms Underlying Diarrhoea

Another AE associated with the use of BTKis is diarrhoea, which is also often related to the inhibition of the EGFR. Based on the summarized data from clinical trials (Table 3), we find that in ibrutinib-treated patients, diarrhoea is reported in 3-65% (0–10% ≥ *grade 3*) (Treon et al., 2018; Munir et al., 2019; Waller et al., 2019). The frequency of diarrhoea is similar during acalabrutinib-treatment, 17–58% of patients develop this AE but the severity seems to be lower, with 0.6-5% of the patients having diarrhoea ≥ *grade 3* (Awan et al., 2019; Byrd et al., 2020b; Ghia et al., 2020; Sharman et al., 2020). In clinical trials, where tirabrutinib was used, diarrhoea was reported in 7-44 % (0–7% ≥ *grade 3*) (Walter et al., 2017; Rule et al., 2020; Sekiguchi et al., 2020).

As mentioned, the results from clinical trials in CLL, direct comparisons of BTKis e.g. ibrutinib versus acalabrutinib, are not available during the time of the writing of this review. However, in a randomized phase 3 trial in symptomatic WM, the results demonstrate that zanubrutinib treatment is associated with a trend toward lower toxicity, including diarrhoea (Tam et al., 2020a). Among zanubrutinib treated patients in the NCT03053440 trial, diarrhoea was reported in 21% of patients in the zanubrutinib arm and in 32% in ibrutinib arm (Tam et al., 2020a). However, on an exposure-adjusted basis, the frequency of diarrhoea among zanubrutinib patients in this trial was only half that reported among ibrutinib patients (1.3 and 2.6 events/100 person-months, respectively). Additionally, the functional scale for diarrhoea trended worse for ibrutinib than zanubrutinib patients, which was consistent with the frequency of diarrhoea reported for each treatment arm. This was attributed to a less potent inhibition of EGFR by zanubrutinib (Tam et al., 2020a). However, if this would be the etiology, our interpretation is that a high frequency of diarrhoea in patients on acalabrutinib- or tirabrutinib would not be expected, since these inhibitors virtually do not inhibit EGFR (Byrd et al., 2016; Liclican et al., 2020).

The BRK/PTK6 kinase negatively regulates growth and promotes enterocyte differentiation in the small intestine, possibly by regulating β-catenin through AKT (Haegebarth et al., 2006). BRK is normally expressed in intestinal cells and is thought to play a role in epithelial barrier function. Suppression of BRK is associated with an increase in apoptosis of proliferating cells (Basile et al., 2019). Inhibition of BRK has been reported for ibrutinib (IC50: 3.3 nM) (Honigberg et al., 2010) and zanubrutinib (IC50: 33 nM) (Guo et al., 2019), however, no cases of stomatitis/mucositis have been reported in acalabrutinib- or zanubrutinib-treated patients (Sibaud et al., 2020). While it is always difficult to estimate an inhibitory effect on kinases not carrying a cysteine in the catalytic cleft, zanubrutinib binds to BRK with a higher IC50 than ibrutinib (Guo et al., 2019). Collectively, based on all of these observations, we do not favour an EGFR off-target effect as the only mechanism underlying the frequent occurrence of diarrhoea in patients treated with BTKis.

## Mechanisms Underlying Increased Incidence of Infections

Increased rates of infectious complications occur in patients treated with ibrutinib. As reported by Tillman et al. (2018) in the systematic review of infectious events summarizing data from analysis of all prospective trials of ibrutinib used in hematologic malignancies, covering 48 trial cohorts, infections of any grade occurred in 56% of patients taking single−agent ibrutinib and 52% of those on combination therapy. *Grade 3−4* infectious AEs occurred in 26% of patients on single−agent studies and 20% of patients receiving combination therapy. The rate of *grade 5* infectious AEs was 2% in both cohorts (Tillman et al., 2018).

In another publication analyzing findings from 378 patients with lymphoid cancer who received ibrutinib during a 5-year period, serious infections were reported in 11%, primarily during the first year of ibrutinib treatment (Varughese et al., 2018). Of interest is the fact that there is an increased risk for opportunistic infections (OI), especially Invasive fungal infections (IFIs) with Aspergillus species being most frequently identified (Ahn et al., 2016; Messina et al., 2017; Ghez et al., 2018; Rogers et al., 2019a).

In the above mentioned retrospective cohort study of 378 patients from Memorial Sloan Kettering, IFI was reported in 4.2% of patients (Varughese et al., 2018). In a single institution retrospective cohort study at the Ohio State University, including all patients who received ibrutinib for the treatment of a hematologic malignancy between 2010 and 2016, 23 cases of OI were reported among 566 patients. The majority of OIs were IFIs, observed in 17 of the patients, and invasive aspergillosis was the most frequent. IFIs occurred at a median of 4 months after starting ibrutinib. In patients receiving ibrutinib as frontline therapy OIs were not increased, but were associated with higher number of previous treatments, suggesting that the cumulative immunosuppressive effects of prior therapies may contribute to the risk (Rogers et al., 2019a). Of interest is also a fact that not all B-cell malignancies show the same susceptibility. In CLL the risk is considerable, whereas in WM the incidence of IFIs seems to be very low (Cheng et al., 2019), suggesting that parameters other than the drug itself contribute, e.g. immune dysregulation from the underlying malignancy. Of note, in a small trial for primary central nervous system lymphoma as many as 39% of patients treated with ibrutinib plus corticosteroids developed aspergillosis (Rogers et al., 2019a).

The mechanism underlying susceptibility to IFIs seems to be complex but a direct effect of ibrutinib on the immune system, mediated by both on- and off-target kinase inhibition, is most probably involved. Both macrophage and neutrophil functions are highly relevant for the anti−fungal immune response, and these cell types simultaneously express BTK and TEC. Neutropenia is a common side effect in patients treated with ibrutinib (Byrd et al., 2020a) and, as mentioned, this could be secondary to the tumor burden in combination with suppressed intracellular signaling. A key role for BTK in macrophage responses during experimental pulmonary aspergillosis has been reported. BTK activation led to calcineurin-NFAT signaling, which was considered crucial for orchestrating neutrophil recruitment during pulmonary aspergillosis (Herbst et al., 2015).

In another study, *A. fumigatus* was shown to induce BTK phosphorylation in human macrophages, while BTK depletion impairs NFAT and NF-κB responses. A TLR9-dependent endosomally driven pathway was implicated to increase the susceptibility of patients on ibrutinib to IFI (Bercusson et al., 2018). A recent report (Fiorcari et al., 2020), shows that ibrutinib- or acalabrutinib-mediated BTK inhibition negatively affects CLL-associated macrophages during *A. fumigatus* infection. It was also reported that ibrutinib and acalabrutinib impaired M1 polarization in macrophages, a phenotype associated with an efficient anti−microbial immune response (Colado et al., 2020). Furthermore, treatment with ibrutinib was shown to reduce the phagocytic ability and increase the immunosuppressive profile of nurse-like cells exacerbating the expression of M2 markers (Fiorcari et al., 2016).

The clinical hallmark of congenital BTK mutations causing XLA is recurrent bacterial infections, but there are also a few reports of OIs such as *Pneumocystis jirovecii* (Jongco et al., 2014). In XLA, the ibrutinib-sensitive TEC kinase is present and may functionally substitute for BTK in non-B-cells. It is therefore possible that the combined inhibition of BTK and TEC may cause the augmented susceptibility to IFI.

Another proposed mechanism for the observed increase in IFI, is the possibility that the risk is secondary to thrombocyte impairment. Thus, among BTKis, ibrutinib is known to negatively affect platelets. Hence, it has been reported that human platelets attenuate Aspergillus species (Perkhofer et al., 2008) and data reported in a recent publication from the same laboratory there is data suggesting that platelets could trigger coagulopathy and activate neutrophils during aspergillosis (Fréalle et al., 2018).

Finally, it could be argued that impairment of the ITK kinase, which also carries a cysteine in its catalytic site, could contribute to the increased incidence of infection, since this kinase is impaired by certain BTKis (Dubovsky et al., 2013; Estupiñán et al., 2020). ITK is expressed in T-cells and it is a critical regulator of the T-cell development and function, including for immune responses to parasitic and viral infections. Thus, humans with congenital ITK deficiency have a predilection for severe viral and opportunistic infections including Candida species, *P. jirovecii*, Epstein−Barr virus, and Varicella zoster virus (Ghosh et al., 2014). ITK inhibition by ibrutinib may therefore contribute to the OIs and the forthcoming trials comparing the extent to which drugs, bind, or not bind, to ITK, will provide information related to this topic.

## Concluding Remarks

The number of drugs inhibiting BTK has been steadily increasing, and, owing to their specific properties they show clinical differences, for instance with regard to AEs. In the case of leukemia and lymphoma treatment, the new BTKis will also make it possible to introduce other binding modes to overcome resistance mutations, since these will vary between e.g. reversibly and non-reversibly binding compounds. We here provide an update on the currently available BTKis, discuss some of their unique properties and present the ongoing trials in table format for a reader-friendly overview. The current 22 BTKis are studied in hundreds of clinical trials, including a large number in phase III.

**FIGURE 1:**
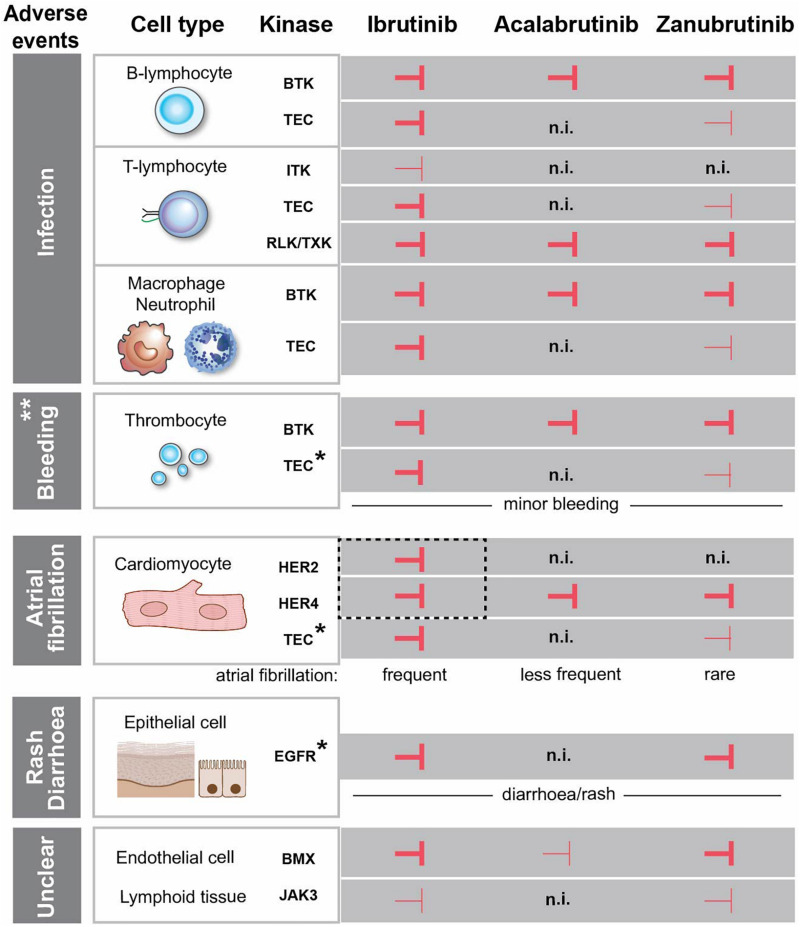
Effects of FDA-approved BTK-inhibitors targeting tyrosine kinases with conserved cysteine. Inhibition of the kinases is depicted with 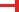 and 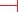 corresponding to strong and weak inhibition, respectively. ** Major bleedings are more frequent upon ibrutinib treatment versus more selective BTKi. *The association between the kinase inhibition and the side effect is not entirely obvious, since the observed adverse event occurs even if the kinase is not inhibited. EGFR is expressed in the epithelial cells in the gut and skin, but we do not favour a direct association between EGFR-inhibition and diarrhoea/rash. The same is the case for TEC, which is expressed in cardiomyocytes and thrombocytes and not inhibited by acalabrutinib, while the corresponding side effects still occur. HER2 and HER4 are in dashed box, since we favour the association of the simultaneous inhibition of these two kinases with atrial fibrillation. (n.i.) no inhibition. Cardiomyocyte and epithelial cells were created with BioRender.com.

We also review the origin of AEs. The lack of EGFR inhibition reported for acalabrutinib in different assays, is difficult to reconcile with the prevailing notion that rash is secondary to inhibition of the EGFR by BTKis. Likewise, we find a lack of compelling evidence demonstrating that off-target EGFR-binding is the main mechanism for diarrhoea, given the major differences in affinity for this receptor among the various BTKis. Instead, this suggests that mechanisms other than binding to kinases carrying a cysteine in the active site contribute to the observed adverse effects. Regarding the augmented incidence of invasive fungal infections during BTKi treatment, the cause has not yet been conclusively identified. However, we favour the involvement of detrimental effects on phagocytes, likely caused by neutropenia induced by the tumor burden as well as by direct effects of BTKis on intracellular signaling components. A similar dual effect on thrombocytes may also explain the increased frequency of bleedings mainly observed during the early phase of BTKi therapy. In contrast, we find that the EGFR-family members ERBB2/HER2-ERBB4/HER4 are strong candidates for involvement in cardiovascular AEs, such as AF. They are expressed in the heart, both carry a cysteine in their catalytic site, permitting ibrutinib binding in both cases. Figure 1 summarizes the treatment- and the adverse effects of the FDA-approved BTKis, namely ibrutinib, acalabrutinib and zanubrutinib. While we have learnt a lot over the recent years, the studies of these inhibitors are derived from patients with B-cell malignancies, which means that it is not possible to separate adverse effects caused only by the inhibition of kinases from indirect effects caused by the specific contribution from the tumor and its environment.

## Author Contributions

HYE and AB collected, analyzed, and wrote the manuscript. RZ evaluated assays for enzyme activity in the presence of inhibitors, participated in discussions and data interpretation and edited the manuscript. CIES perceived and conceptualized the idea and was involved in data interpretation and writing the manuscript. All authors contributed to the article and approved the submitted version.

## Conflict of Interest

The authors declare that the research was conducted in the absence of any commercial or financial relationships that could be construed as a potential conflict of interest.
